# Luxation trans-scapho-lunaire associée à une fracture-luxation verticale de pyramidal: un cas très rare

**DOI:** 10.11604/pamj.2016.23.138.8751

**Published:** 2016-03-26

**Authors:** Hmouri Ismail, Mohamed Amine Karabila

**Affiliations:** 1 Service de Chirurgie Traumato-orthopédie, CHU Ibn Sina, Rabat, Maroc

**Keywords:** Luxation, fracture, pyramidal, Dislocation, fracture, pyramid

## Image en médecine

Nous rapportons le cas d'un jeune homme de 21 ans victime d'une chute de sa moto sur la main droite occasionnant une fracture luxation trans-scapho-rétro-lunaire associée à une fracture verticale de pyramidal (A et B). A j + 2 le patient a bénéficié d'une réduction sanglante (C) des os du carpe désorganisés avec ostéosynthèse du scaphoïde et du pyramidal (D). La fracture-luxation péri-lunaire est une lésion rare constituant 5% à 10% des lésions traumatiques du poignet, souvent sous-diagnostiquée et nécessite un traumatisme en hyperflexion palmaire, l'association d'une luxation-fracture de pyramidal est encore plus rare. Les luxations péri-lunaires du carpe peuvent laisser des séquelles fonctionnelles graves si elles ne sont pas diagnostiquées rapidement après le traumatisme. Le diagnostic est très aisé et un simple examen radiologique permet de poser le diagnostic.

**Figure 1:**
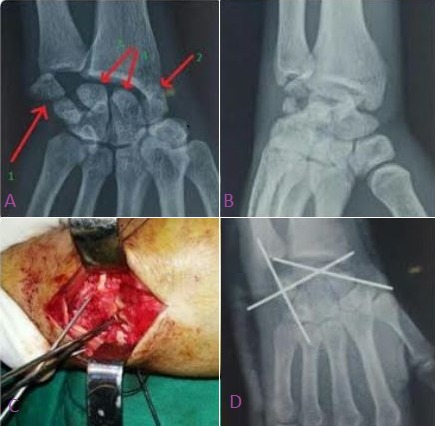
(A) incidence de face: 1) fracture luxation verticale de pyramidal, 2) fracture de scaphoïde, 3) ascension du capitatum qui a pris la place de semi-lunaire, 4) luxation de semi-lunaire; (B) incidence trois quart du poignet montrant la désorganisation complète du poignet; (C) vue peropératoire après réduction et embrochage; (D) contrôle radiologique: bonne congruence articulaire

